# High egg rejection rate in a Chinese population of grey-backed thrush (*Turdus hortulorum*)

**DOI:** 10.24272/j.issn.2095-8137.2019.039

**Published:** 2019-05-18

**Authors:** Can-Chao Yang, Long-Wu Wang, Wei Liang, Anders Pape Møller

**Affiliations:** 1 Department of Neurology, Army Medical Center of PLA, Chongqing 400042, China; 2 State Forestry Administration of China Key Laboratory for Biodiversity Conservation in Mountainous Areas of Southwest Karst, School of Life Sciences, Guizhou Normal University, Guiyang Guizhou 550001, China; 3 Ecologie Systématique Evolution, Université Paris-Sud, CNRS, AgroParisTech, Université Paris-Saclay, F-91405 Orsay Cedex, France

## DEAR EDITOR,

Several previous studies have indicated that nest sanitation behavior is a general adaptation in altricial birds, with egg recognition capacity evolving as a specific response to interspecific brood parasitism (IBP). However, a recent study suggested an alternative hypothesis, concluding that conspecific brood parasitism (CBP) selects for egg rejection in thrushes, with IBP as a by-product. In the present study, we used a spectrophotometer to quantify egg coloration and egg mimicry and performed artificial parasitism experiments in the grey-backed thrush (*Turdus hortulorum*). We showed that individuals of this species rejected 100% of 12 foreign eggs, without IBP or CBP detected. In a review of previous studies, we also discuss possible explanations for the high egg rejection rate in the grey-backed thrush and suggest areas for future study.

Altricial birds have evolved advanced reproductive behavior to increase the fitness of their offspring by building elaborate structures (i.e., nests), in which they lay eggs and rear their nestlings (Hansell, 20007). Bird nests not only provide a suitable place for nestling development, but also act as a concealed location for safety from predators. Furthermore, bird parents have evolved nest sanitation behavior to clean foreign objects from their nests, including feces, eggshells, branches, and leaves, because they induce predation, facilitate microorganism growth, damage eggs, or hurt nestlings during brooding (Guigueno & Sealy, 2012). Therefore, nest sanitation has evolved as a general behavior in altricial birds for distinguishing between egg-shaped and non-egg-shaped objects. This recognition capacity has further improved in some species to facilitate detection of differences within eggs (i.e., egg recognition) as a specific adaptation to avian brood parasitism, where other birds lay parasitic eggs in nests that are not their own, thereby reducing reproductive output of the hosts (Davies, 2011; Yang et al., 2015a). Avian brood parasitism can be classified as either interspecific brood (IBP) or conspecific brood parasitism (CBP). Numerous empirical studies have shown that IBP selects for the capacity of hosts to recognize and reject foreign eggs (Davies, 2000; Liang et al., 2016; Moksnes et al., 1991; Rothstein & Robinson, 1998; Yang et al., 2010). Alternatively, the “collateral damage” hypothesis states that CBP is responsible for egg rejection in birds, with rejection due to IBP constituting a by-product of host adaptations against CBP (Jackson, 1998). However, this hypothesis failed to explain egg recognition by hosts because it was tested and supported in a single non-passerine bird species (Lyon & Eadie, 2004, 2008). Recently, new research re-examined this hypothesis and drew supportive conclusions by testing it in two passerine species of thrush, that is, the song thrush (*Turdus philomelos*) and European blackbird (*Turdus merula*) (Samas et al., 2014a). However, Soler (2014a) stated that, to date, there is no evidence of CBP causing egg rejection in thrushes *per se*, though Samas et al. (2014b) subsequently supported their conclusion with empirical evidence. Similarly, Ruiz-Raya et al. (2016) investigated recognition of conspecific or heterospecific eggs in European blackbirds by manipulating the risk of CBP and IBP, respectively. They found that blackbirds presented low recognition of conspecific eggs even under high risk of CBP, and thus their results supported the IBP hypothesis that egg recognition has evolved and is maintained in blackbirds as a response to previous cuckoo parasitism.

Here we performed an empirical study to test egg recognition capacity in the grey-backed thrush. The main purpose of this study was to provide initial information on egg recognition in this species, which may facilitate further study. According to previous studies on *Turdus* spp., we predicted that the grey-backed thrush would not show egg recognition capacity because no CBP or IBP has been found in this population. An alternate prediction was also considered, that the grey-backed thrush may also display egg recognition due to previous IBP by cuckoos, which still affects host behavior.

**Figure 1:**
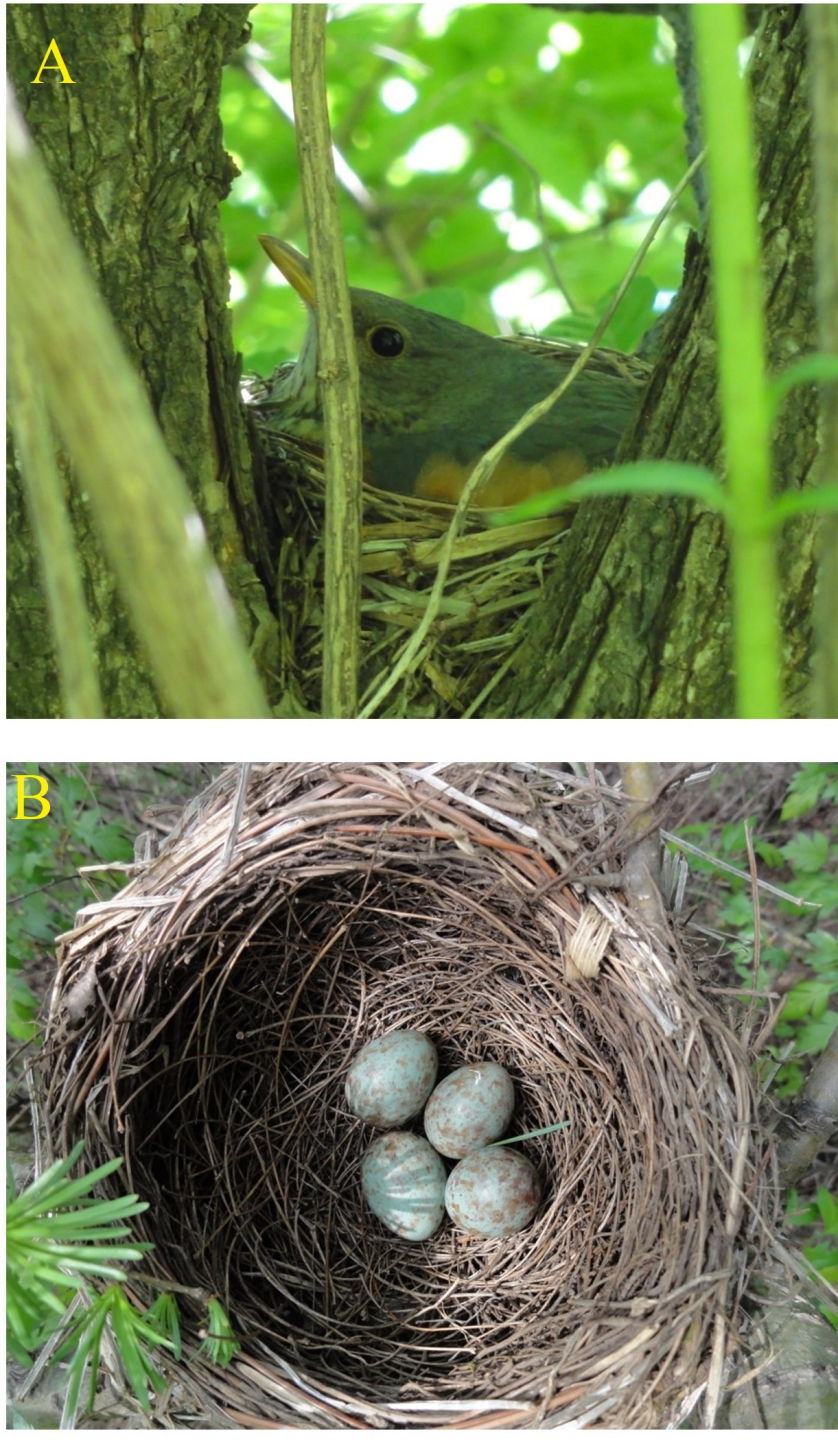
Nest site, nest, incubating female, and eggs of the grey-backed thrush (Photos by Long-Wu Wang)

This study was performed in Fusong County, Jilin Province, China (N42º 19' 382'', E127º 15' 107''), an area of secondary forest fragmented by corn (*Zea mays*) crop farmland and scattered plantations (dominated by larch *Larix* spp.), from May to June 2013. This region is in the temperate zone at an elevation of 481 m, with a continental monsoon climate characterized by cold and snowy winters with an average annual temperature of 4 ºC. The grey-backed thrush belongs to the Turdidae family and is mainly distributed in East Asia (MacKinnon & Phillipps, 1999), where it chooses nest sites with short ground cover and a high density of small trees and shrubs (Zhou et al., 2011). In our study site, open-cup nests were built in trees (Figure 1A), and pale green eggs with reddish markings were laid in these nests. (Figure 1B), with an average clutch size of 4.42 eggs±0.51 (range 4–5 eggs, *n*=12).

All experiments complied with the current laws of China. Experimental procedures were in accordance with the Animal Research Ethics Committee of Hainan Provincial Education Centre for Ecology and Environment, Hainan Normal University (permit No. HNECEE-2012-004).

The appearances of the thrush and model eggs were quantified with a spectrophotometer (Avantes-2048, Avantes, Apeldoorn, The Netherlands). Six reflectance spectra were measured in each egg and averaged to represent the color of the egg. Model eggs were immaculate blue, a common coloration of cuckoo eggs in China (Yang et al., 2010, 2012). Thrush eggs are pale green with reddish markings and thus their egg ground color and markings were measured, respectively. Subsequently, egg spectra were loaded into AvaSoft 7.3, at which the wavelength range of spectra varied from 300 to 700 nm. The spectral range of 300–400 nm and 400–700 nm refers to ultraviolet (UV) light and visible light (VIS), respectively (for details, see Yang et al., 2010, 2013).

**Figure 2:**
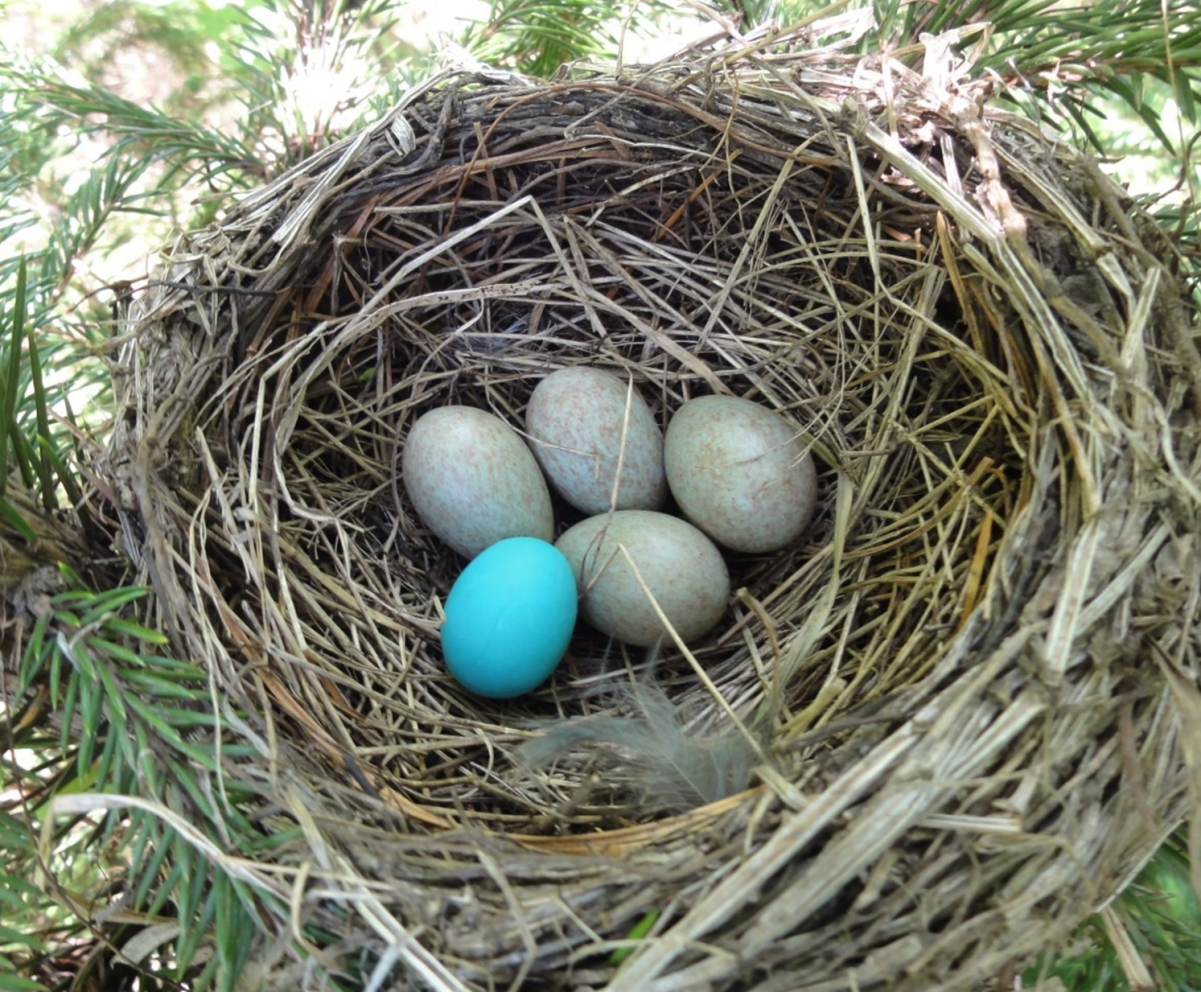
Experimental nest of the grey-backed thrush with a blue model egg (Photo by Long-Wu Wang)

Nests of the grey-backed thrush were found by searching potential nest sites and monitoring the activity of reproductive adults. Nests were then randomly sorted into two groups: (1) manipulated group into which one immaculate blue model egg was introduced just after clutch completion (*n*=12) (Figure 2); and (2) control group in which nests were visited by the same procedure to control for human disturbance, but no manipulation was made (*n*=10). Manipulation was performed in these circumstances without observing hosts to avoid potential effects of host observations on recognition (Hanley et al., 2015). Observed nests were monitored for 6 d after manipulation and the responses of thrushes to foreign eggs were classified as rejection, if foreign eggs were ejected, pecked, or deserted, or accepted if foreign eggs were intact and incubated (Yang et al., 2010, 2014b). Model eggs were made by polymer clay and their sizes were standardized to 25 mm×19.5 mm, similar to thrush eggs (25.07±0.42 mm×19.74±0.71 mm, *n*=10).

**Figure 3:**
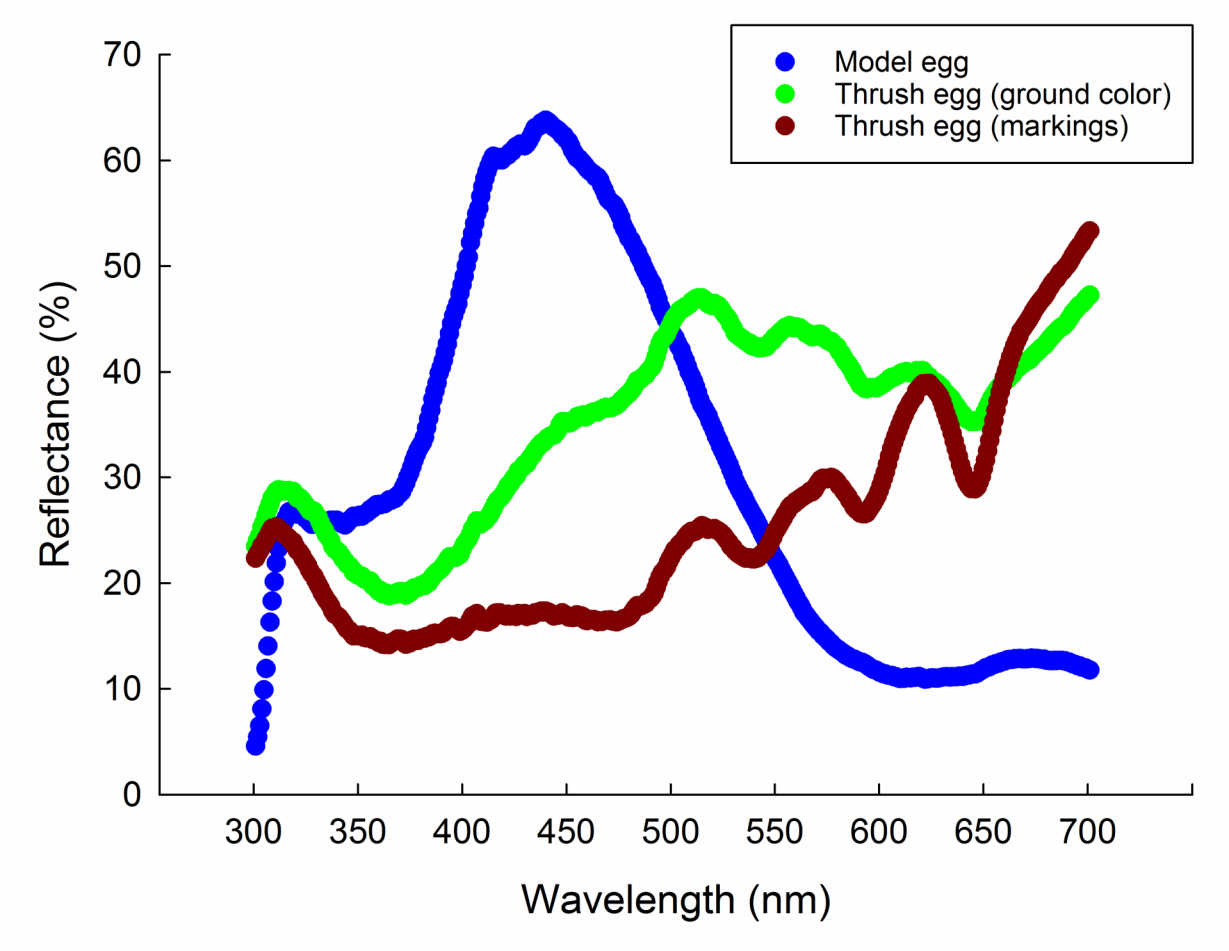
Egg reflectance of the grey-backed thrush and model eggs

The grey-backed thrushes laid pale green eggs with dense reddish markings (Figure 1B). Egg reflectance analysis illustrated that egg ground color was consistent with human eye assessment, with a reflectance peak in the range of green light (475–550 nm) (Figure 3). Similarly, the reddish markings had a reflectance peak in the range 550–620 to 620–700 nm, which represents yellow and red light, respectively (Figure 3). The blue model egg had a reflectance peak in the range of blue light (400–475 nm) (Figure 3). The reflectance contrast between trough and peak reflects chroma (or color saturation). Therefore, blue model eggs were more saturated in color than the thrush egg. In brief, the model egg was very different from thrush eggs according to vision based on human eyes and spectral reflectance. Experimental parasitism indicated that the grey-backed thrush rejected all non-mimetic model eggs, with a rejection rate of 100% (*n*=12). All rejections occurred within a day (i.e., 24 h) and were all performed by ejection without any recognition error. No rejection was found in the control group (*n*=10).

This Chinese population of grey-backed thrush possessed high recognition capacity, rapidly rejecting 100% of non-mimetic foreign eggs. This contradicted our expectation that grey-backed thrush should have no or low-level egg recognition capacity because no CBP or IBP was detected in our study population. Thus, this egg rejection ability of the grey-backed thrush needs further investigation.

A recent study on artificial parasitism in song thrushes and European blackbirds found unusually high rejection rates of CBP (up to 60%) and positive co-variance with conspecific population densities without risk of IBP (Samas et al., 2014). Because IBP rejection rates did not covary geographically with IBP risk (Grim & Stokke, 2016) and thus contradicted the IBP hypothesis that egg recognition has evolved as a response to IBP, Samas et al. (2014) concluded that egg recognition in *Turdus* spp. has evolved as a response to CBP, not to IBP. However, if egg rejection abilities can be maintained in the absence of IBP, high egg rejection rates can still be retained without geographic co-variation. Therefore, Soler (2014a) criticized the conclusion of Samas et al. (2014) and argued they made an invalid conclusion due to an out-of-date theoretical background and a biased selection of references. However, Samas et al. (2014b) argued that a theory is never out of date and addressed the theoretical objections by empirical evidence. Recently, Ruiz-Raya et al. (2016) further tested egg recognition in blackbirds by manipulating the risk of CBP and IBP and concluded that selection from IBP likely accounts for egg recognition in blackbirds.

In our study population, grey-backed thrushes displayed high recognition capacity of foreign eggs. It is generally accepted that IBP rather than CBP contributes to egg recognition in hosts. Firstly, egg recognition capacity is much more unlikely to evolve in response to CBP than IBP because IBP gives rise to dramatic fitness costs, which are much lower than those from CBP (Petrie & Møller, 1991). Parasites and hosts with CBP are conspecific and share the same gene pool and thus constitute much weaker selection than IBP (Ruiz-Raya et al., 2016; Soler, 2014b). Furthermore, conspecific egg phenotypes in CBP are too similar to initiate evolution of egg recognition (Soler et al., 2011). However, intraspecific variation in egg coloration is high for some host species, and CBP may account for the evolution of egg recognition (Cassey et al., 2008a, 2008b; Hanley et al., 2017; Samas et al., 2014a). Secondly, in order to select for conspecific egg rejection, the level of CBP must be high. However, in our study population, no CBP was detected. Similarly, Samas et al. (2014) described that the rates of CBP were only 0%–2.2% and 0%–3.1% for the song thrush and blackbird, respectively. However, current parasitism rates may not represent actual selection pressure from IBP and CBP without considering other factors, such as recognition error and rejection cost. Furthermore, because egg rejection capacity has evolved in response to IBP, and it can be maintained in the absence of IBP, this may also occur in response to CBP but in its absence. Many currently unparasitized potential host species exhibit a rejection rate of nearly 100% (Lahti, 2006; Peer & Sealy, 2004; Yang et al., 2014a, 2015b). For example, blackbirds were introduced in the nineteenth century to New Zealand, where a high rejection rate of non-mimetic eggs has been reported (62%, Samas et al., 2014; 83.9%, Hale & Briskie, 2007), similar to the rejection rate of 90% in Europe (Grim et al., 2014; Martín-Vivaldi et al., 2012; Moskát et al., 2003). Moreover, a recent review concluded that it is not correct to formulate predictions that assume that rejection behavior of hosts must be lost in the absence of obligate brood parasites (Soler, 2014b). Finally, aggressive behavior towards adult cuckoos and reluctance to feed cuckoo chicks has been empirically shown in thrushes (Grim et al., 2011), providing evidence for contact with IBP in the past, resulting in successful resistance against interspecific brood parasitism (Ruiz-Raya et al., 2016). However, Samas et al. (2014b) argued that aggression in blackbirds did not specifically evolve in response to IBP because they are aggressive not only to cuckoo dummies but also to any intruders near their nests, including harmless pigeons (*Columba livia*). Thus, switching to new types of food is an unlikely defense against brood parasite chicks because such changes would not prevent most costs from IBP (Grim et al., 2011; Samas et al., 2014b). However, Ruiz-Raya et al. (2016) found that blackbirds were able to recognize and eject heterospecific eggs at high rates, whereas most conspecifics eggs were not recognized. Moreover, ejection rates of conspecific eggs did not exceed 13%, even in the presence of a high risk of CBP, whereas ejection rates of experimental eggs simulating IBP were much higher (80%–100%). Female blackbirds were also found to be more aggressive towards cuckoos than towards blackbird dummies (Ruiz-Raya et al., 2016). Additionally, Ruiz-Raya et al. (2016) estimated that the level of CBP necessary to select for evolution of host response against conspecific eggs would range from 55% to 65%. Because the grey-backed thrush has retained a high level of egg recognition, the rejection costs, which would contradict such maintenance, should be negligible. According to our results, no rejection cost was detected. Like blackbirds, the grey-backed thrush also has a large bill to grasp foreign eggs for rejection. Therefore, rejection costs should not prevent grey-backed thrushes from retaining egg rejection capacity.

In summary, previous studies on thrush hosts have provided inconsistent conclusions. This situation is complicated by the explanation for one species not being suitable for another. Our study provides preliminary information, and thus cannot offer sufficient evidence to support either the IBP or CBP hypothesis. However, considering that previous studies have provided strong evidence that hosts affected by IBP can retain egg recognition capacities after long-term escape from cuckoo parasitism (Lahti, 2006), egg rejection capacity in grey-backed thrushes may be maintained because parasitic cuckoos have exploited this potential host in the past. Although theoretically hosts may also retain egg recognition due to previous CBP, like IBP, empirical studies are currently insufficient to clarify this assumption. Furthermore, recent studies have revealed that egg accepters can become rejecters after stimulation, in all cases switching from acceptance to rejection, implying that historical cases of IBP or CBP are considerably underestimated in currently non-parasitized potential host species (Molina-Morales et al., 2014; Yang et al., 2015b). Further studies referring to egg color variation and recognition with different degrees of egg mimicry are necessary in the grey-backed thrush and even other species of *Turdus*. Such studies will help us better understand the origin of egg recognition in thrushes.
